# Supplementary figures and images for: Free ISG15 inhibits Pseudorabies virus infection by positively regulating type I IFN signaling
Source: PLoS Pathog. 2022 Oct 31;18(10):e1010921. doi: 10.1371/journal.ppat.1010921 (PMC9648840; doi:10.1371/journal.ppat.1010921)

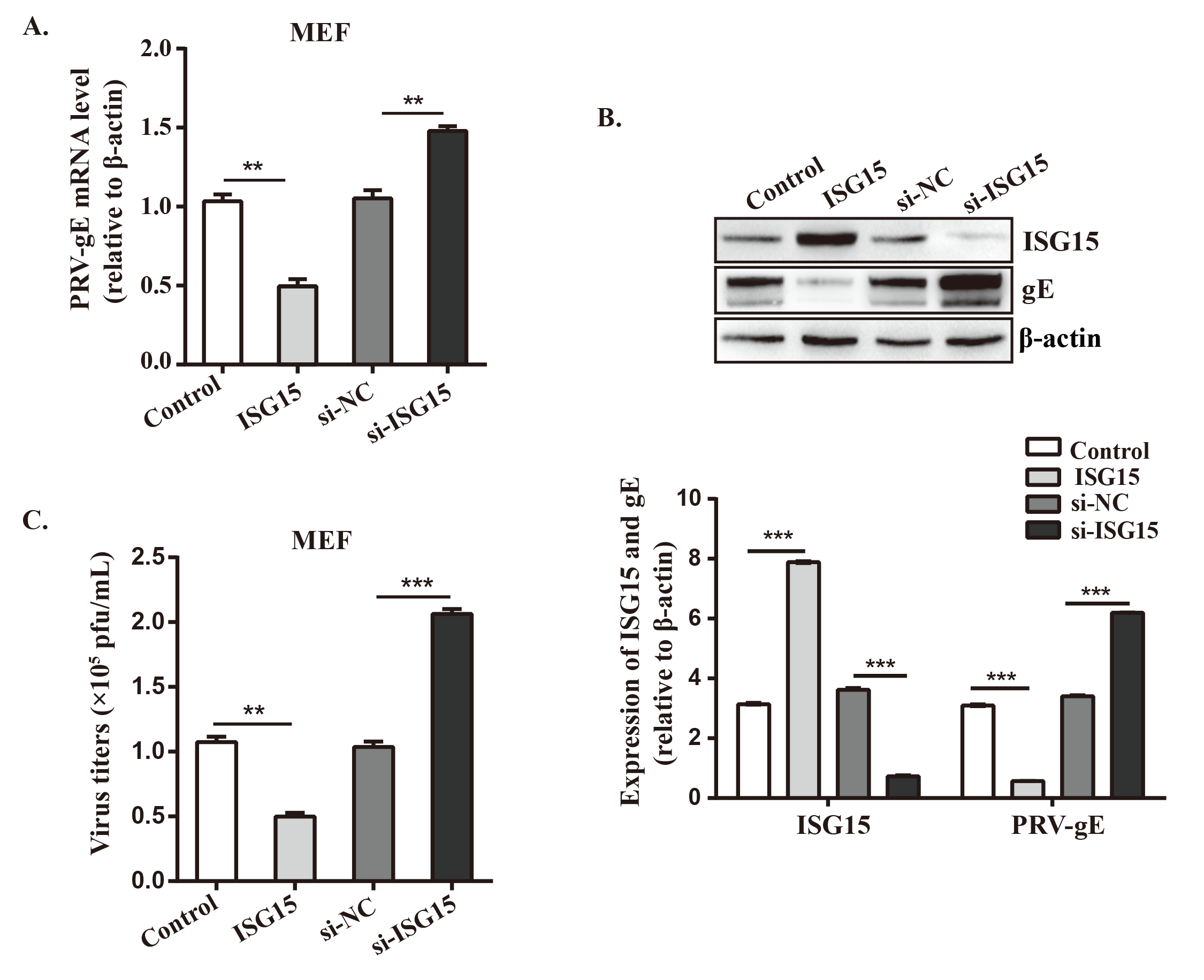

Supplement: S1 Fig — (A to C) MEF cells were transfected with empty-vector, or a plasmid expression expressing ISG15, or control siRNA or ISG15 siRNA, and then infected with PRV (MOI = 1) for 24 h. The mRNA and protein expression of PRV-gE, and PRV titer were detected by RT-qPCR, Western blot and plaque assays respectively. Each experiment was repeated at least three times separately. *, p < 0.05; **, p < 0.01; ***, p < 0.001 (t-test). (TIF) [file ppat.1010921.s001.tif]

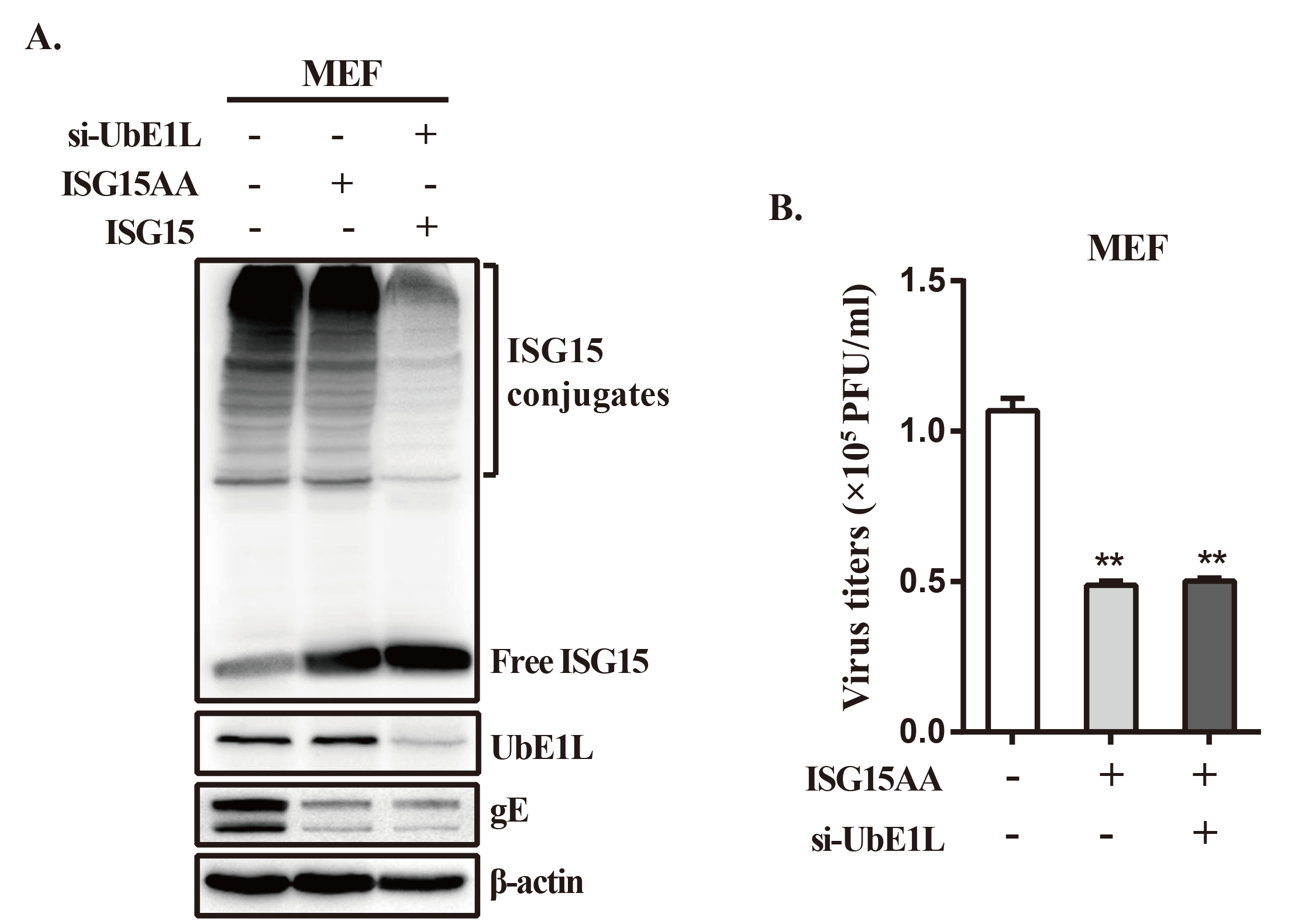

Supplement: S2 Fig — (A and B) MEF cells were transfected with UbE1L siRNA, or ISG15AA, or ISG15, and then were infected with PRV. The expression of PRV-gE and ISG15 and PRV titer were detected by Western blot and plaque assays, respectively. **, p < 0.01 (t-test). (TIF) [file ppat.1010921.s002.tif]
