# Supplementary material for: Free ISG15 inhibits Pseudorabies virus infection by positively regulating type I IFN signaling
Source: PLoS Pathog. 2022 Oct 31;18(10):e1010921. doi: 10.1371/journal.ppat.1010921 (PMC9648840; doi:10.1371/journal.ppat.1010921)
Supplement: Attachment — Submitted filename: response to reviewers.docx [file ppat.1010921.s003.docx]

**Responses to Reviewers**

Dear Eain:

On behalf of all the contributing authors, I would like to express our sincere appreciations of your letter and reviewers’ constructive comments concerning our article entitled “Free ISG15 inhibits Pseudorabies virus infection by positively regulating type I IFN signaling (Manuscript No.: PPATHOGENS-D-22-01388)”. These comments are all valuable and helpful for improving our article. According to the editor and reviewers’ comments, we have made extensive modifications to our manuscript and supplemented extra data to make our results convincing. Those changes are tracked in the revised manuscript. Here we are addressing the reviewers’ comments and concerns in detail are listed below this letter.

**Reviewer #1**

In this manuscript, the authors present a molecular mechanism of ISG15-mediated antiviral response against pseudorabies virus. Viruses trigger robust IFN response in infected cells, and IFN via IFN-induced proteins, called ISGs, mediates the antiviral protection in infected and uninfected cells. In this study, the authors examined how ISG15, a potent antiviral ISG, exerts its antiviral effects using a novel, previously unknown mechanism. ISG15 is known to function by conjugating with target proteins; however, the current study suggests that ISG15 can also function by a non-conjugation mechanism. Pseudorabies infection caused robust induction of ISG15, tested by complementary approaches, in PK15 cells. ISG15 KO cells showed enhanced viral replication and reduced IFN production. Using conjugation-defective ISG15 mutant, the authors elegantly showed that the antiviral function of ISG15 was not dependent on conjugated proteins. Finally, they showed that ISG15 promotes IFN-signaling by supporting STAT1-STAT2 complex formation and nuclear translocation. The physiological relevance of the study was examined in ISG15 KO mice, which were more susceptible to virus infection due to increased viral load and neuronal and pulmonary inflammation. Overall, the study is well-designed and uses complementary approaches and appropriate controls. Some weaknesses, as pointed out below, may help improve the study.

Re: We appreciate reviewer’s comments on the importance of this study. According to reviewer’s advice, we amended the relevant part in manuscript. Here below is our description on revision according to the reviewer’s comments.

1. The entire study was performed in one cell line, PK15, although it is unclear which cells were used for the ISG15 KO studies. The figure legends only show Wt and KO cells, but no details are provided.

Re: We agree with the comment and have provided the details about the WT and KO cells in the revised manuscript as following: the wild type PK15 (WT) and ISG15 deletion in PK15 cells (ISG15^-/-^) cells were used to study the role of ISG15 during PRV infection.

2.The pathogenesis study was done in mice without testing whether the phenomenon or the mechanism would be valid in the mouse system. Is the conjugation-independent ISG15 mechanism functional in mouse cells as well?

Re: We accepted your comments and added some experiments in Fig 2. The results showed that ISG15 overexpression inhibited PRV replication, whereas knockdown of ISG15 facilitated PRV replication in mouse embryonic fibroblast (MEF) cells, further supporting the antiviral role of ISG15. Moreover, the antiviral role ISG15 via conjugation-independent was also confirmed in MEF cells.

3. There is a disconnect between the role of ISG15 in IFN production and IFN signaling. The IFN-signaling part has been followed up by testing STAT1-STAT2 complex formation. However, how ISG15 functions in the IFN production pathway is not known.

Re: We have added extra experiments and identified that ISG15 facilitated type I IFN production by activating IRF3. The productive IFNs bind to the receptors and activate type I IFN signaling pathway, which in turns induces ISG15 expression. ISG15 serves a positive role in IFN-mediated antiviral response by facilitating IFN production and IFN signaling.

4. For the mechanistic studies, it is unclear whether ISG15 interacts with either STAT1 or STAT2 or both. How unconjugated ISG15 promotes complex formation was not studied. Does ISG15AA interact with the STATs?

Re: We are grateful for the suggestion. The experimental data on whether ISG15 or ISG15AA interacts with either STAT1 or STAT2 has been added in the revised manuscript. The results showed that ISG15 could interact with STAT2, rather than STAT1.

5. Fig 6C-E, it is unclear which blots indicate pY-STAT; please label appropriately. Also, the nuclear translocation image is not very convincing in Fig 7D.

Re: Sorry for our mistake, the bolts should be labeled the p-STAT1 or p-STAT2. In the Co-IP experiment, We have revised them in the Fig 6C-E. Also, the nuclear translocation image has been deleted in the revised manuscript.

**Reviewer #2:**

In this manuscript, Liu et al report that ISG15 expression is functionally linked to PRV replication. They found that ISG15 induction by PRV is MOI and time dependent as measured by Western blot, IF ad qPCR analyses. Of note, ISG15 knockout led to increased replication of PRV (about 2to 3-fold). This was attributed to the activity of free ISG15 as opposed to the conjugated one. In a mouse infection model, ISG15 deficiency was mirrored by more severe pathology and rapid death upon challenge with PRV. This in vivo phenotype is a strength of described work. There are also two observations on ISG15 connected to type I IFN production and STAT signaling mediated by IFN. While such results are intriguing, large gaps exist. This is quite evident, especially at the mechanical level where additional evidence is required. Overall, this study suggests a potential relation of ISG15 and PRV replication. Major concerns need to be addressed, which include, missing links, marginal effects of ISG15 (in infected cells), relevance of MOI, gE and data interpretation.

Re: We feel great thanks for your professional review work on our article. As you are concerned, there are several problems that need to addressed. In this study, we found that ISG15 not only facilitated type I IFN production, but also promoted IFN-mediated antiviral response against PRV. This suggests ISG15 serves as a positive regulator in IFN-mediated antiviral response. The induced IFN binds to receptors activates the IFN pathway, and subsequently induced ISG15 expression. According to your nice suggestions, we have made extensive corrections in our revised manuscript.

1. As it stands, Figure 1 shows the expression of ISG15 in response to PRV infection. While there is a dynamic change, it is less clear why the level of ISG15 is reduced panel B). If gE is responsible, what does it do? Additional work will be required. Also, data in panel F seem to be hard to interpret.

Re: The levels of free ISG15 and ISG15 conjugates at high MOI (10) were much lower than those at low MOIs (Fig 1A to 1C), and the decline in ISG15 expression was correlated with increased abundance of a representative viral late protein, gE, by 12h and 24h. This suggested that the high levels of ISG15 present in the cells to prevent PRV growth, however, when cells were infected with a high MOI (10), high level of PRV-gE reduced ISG15 expression. This suggests gE is responsible for ISG15 expression.

To further identify the role of gE in inducing ISG15 expression, we supplemented the extra data in the revised manuscript. As shown in Figs 1E to 1G, gE deletion facilitated free ISG15 expression, but had little effect on ISG15 conjugates. Also, gE overexpression significantly inhibit free ISG15 induction in a dose-dependent manner.

2. ISG15 marginally reduced PRV replication (about 2-fold) as measured by plaque assay (Fig 2A). What is the limit of detection? There is no information on if data is from single or multiple experiments, which also appears a problem throughout the manuscript. If ISG15 inhibits viral release (Fig. 2E), how does it occur?

Re: We feel sorry for our careless mistakes. Thank you for your reminder. The infectivity of each sample was assayed by plaque assay. The mean change in 10^5^ titer ± standard deviation (SD) of three replicates of a representative experiment is shown. The limit of detection in our experiment is 20 pfu/mL. We have added this information in the materials and figure legends in the revised manuscript.

Also, we only found the phenomenon that ISG15 inhibit PRV release. The mechanism of how it occurs is complex, and it would have been interesting to explore this aspect in our further research.

3. Figure 4 indicates a role of ISG15 in the induction of type IFN by PRV. Is it a direct or indirect effect? Something is missing.

Re: The role of ISG15 in the induction of type I IFN by PRV is indirect. To determine if ISG15 is a factor involved in the type I IFN-mediated antiviral response, we firstly examined the effect of ISG15 on IFN production. We have supplemented extra experiment and found ISG15 facilitated type I IFN production by promoting IRF3 activation (Fig 4A).

4. Figure 5 shows a role of ISG15 in the antiviral effect mediated type I IFN in the range of 0.5 to1-fold (panel B and C). Again, it is unclear what is the limit of detection. Figure 6 shows a requirement of ISG15 in the STAT1/STAT2 interaction. Does ISG15 serve as a scaffold? Additional data will be helpful.

Re: Figure 5 shows the change of virus titers is in the range of 1 to 2.5-fold, and the limit of detection in our experiment is 20 pfu/mL. We have added this information in the materials and figure legends in the revised manuscript.

Additional data have been supplemented in Figure 7. The results showed that ISG15 was able to interacted with STAT1, not STAT2.

5. Experimental data in the manuscript are solely derived from a single cell type. Corroboration with other cells will strengthen the authors’ conclusions. Moreover, the gE null virus should be included for viral growth and mouse infection analyses. The manuscript is not clearly written, with typos, errors, and confusing statements (for example, lines 10-11, pp; lines 7-13, pp6).

Re: Thank you for this suggestion. The antiviral role of free ISG15 was confirmed in Mouse embryonic fibroblast (MEF) cells, further strengthening our conclusion. Additionally, animal experiments were used to further validate the antiviral activity of ISG15 against PRV. However, gE is the major virulent gene of PRV and the virulence of gE null virus infection would be attenuated.

We sincerely thank the reviewer for careful reading. We tried our best to improve the manuscript and made some changes to the manuscript.

**Reviewer #3**

The manuscript by Liu et al. investigates the mechanism by which ISG15 inhibits the alphaherpesvirus, pseudorabies virus (PRV), infection. The authors previously demonstrated that PRV infection increased ISG15 expression and that its overexpression was sufficient to reduce viral replication. However, the mechanism by which ISG15 exerted antiviral activity remained unexplored. Here, using a combination of in vitro and in vivo PRV infections the authors argue that the ISG15 monomer (i.e., unconjugated) restricts PRV infection through its role in facilitating phosphorylation of STAT1 and STAT2. Overall, the manuscript addresses an interesting and important question, that is, what the mechanism of ISG15 antiviral activity during PRV infection is. However, the manuscript falls short from supporting this conclusion. In its current state, there are several severe deficits that make interpretations of its data difficult, including a lack of important controls and quantifications, and general difficulties in reading. Below are several suggestions that will help strengthen the manuscript

Re: We sincerely thank the reviewer for your valuable feedback that we have used to improve the quality of our manuscript. According to your nice suggestions, we have added some experiments to support our conclusion, and checked the manuscript and corrected the errors accordingly.

1. Figure 1D: Use of gE has a marker for assessing neighboring cell infection is not useful as UV inactivation results in no detectable gE expression. Moreover, from the images it appears that most cells are infected with WT PRV, thus it is also not possible to tell whether ISG15 is induced in a neighboring cell.

Re: We sincerely appreciate this comment and accept your advice. Figure 1D only shows the ISG15 abundance was associated with viral gene expression.

2. Comments regarding gE being required for the induction of ISG15 are unwarranted. In the gE deleted virus there is still robust ISG15 expression.

Re: From the results of figs 1A to 1D, we hypothesized that gE is responsible for ISG15 expression. To validate this hypothesis, we added some experiments and found that PRV-gE is required for the induction of free ISG15 (Fig 1F to 1G). Results showed that free ISG15 expression gradually increased with increasing infection time when gE of PRV is deleted. Moreover, the overexpression of gE significantly reduced the induction of free ISG15, but no effect on ISG15 conjugates expression. Therefore, we concluded that gE is required for the induction of free ISG15.

3. Figure 2D a control should be added to show the complement of ISG1-/-

Re: Fig 4D showed the expression of PRV-gE in ISG15 complement experiments, while Fig 2 suggested that ISG15 inhibited PRV replication. The graph of Fig 2D showed that the ration of ISG15 deletion (or ISG15 transfection) vs control, and PRV-infected PK15 cell serves as a control.

4. In the text, it is written that fig 2E supernatant has a 3-fold reduction which that graph does not appear to agree with.

Re: Maybe I didn’t clearly express it. The 3-fold reduction in virus titer indicated the ratio of the supernatant of ISG15-transfected cells with respect to control-transfected to the cell-associated of ISG15-transfected cells to control-transfected.

5. Fig 3C should include the expression levels of UbeIL to show how efficient the UbeIL knockdown was

Re: Thanks for your advice. The expression levels of Ube1L in Fig 3C has been added in the revised manuscript.

6. There are multiple typos and grammatical errors throughout the paper

Re: We sincerely thank you for careful reading. We have carefully checked the manuscript and corrected the errors accordingly.

7. Conclusions surrounding 7A are not justified as the STAT1 IP is less efficient. Thus, taking into account IP efficiency, it appears that the same amount complex is IP’d.

Re: Based on your comment, we refined experiment protocols and remade it. Result confirmed that ISG15 deficiency impairs the formation of ISGF3 heterotrimers in Fig 7A.
